# Failure Behavior of Unidirectional Composites under Compression Loading: Effect of Fiber Waviness

**DOI:** 10.3390/ma10080909

**Published:** 2017-08-05

**Authors:** Swaroop Narayanan Nair, Aravind Dasari, Chee Yoon Yue, Srikanth Narasimalu

**Affiliations:** 1 Energy Research Institute at NTU/Interdisciplinary Graduate School, Nanyang Technological University, block S2-B3a-01, 50 Nanyang Avenue, Singapore 639798, Singapore; SWAROOP001@e.ntu.edu.sg; 2 School of Mechanical and Aerospace Engineering, Nanyang Technological University, Singapore, 50 Nanyang Avenue, Singapore 639798, Singapore; MCYYUE@ntu.edu.sg; 3 School of Materials Science & Engineering, Nanyang Technological University, 50 Nanyang Avenue, Singapore 639798, Singapore; 4 Energy Research Institute at NTU, Nanyang Technological University, Cleantech One, 1 Cleantech loop, #06-04, Singapore 637141, Singapore; NSRIKANTH@ntu.edu.sg

**Keywords:** wind turbine blades, defects, laminates, in-plane fiber waviness, compressive strength, kink-band, failure, numerical and experimental analyses

## Abstract

The key objective of this work is to highlight the effect of manufacturing-induced fiber waviness defects on the compressive failure of glass fiber-reinforced unidirectional specimens. For this purpose, in-plane, through-thickness waviness defects (with different waviness severities) are induced during the manufacturing of the laminate. Numerical and experimental results show that the compressive strength of the composites decreases as the severity of the waviness defects increases. A reduction of up to 75% is noted with a wave severity of 0.075. Optical and scanning electron microscopy observations of the failed specimens reveal that kink-bands are created in the wavy regions and lead to failure.

## 1. Introduction

Compressive strength of composite laminates is one of the major design drivers in wind turbine industry. Compressive failure in these materials is complex, sudden and catastrophic as it involves multiple modes of failure like fiber kinking, splitting, buckling and delamination [1,2,3,4]. In 1965, Rosen [1] attributed compressive failure to elastic instabilities resulting in a fiber-buckling mechanism. However, subsequent studies have concluded that compressive failure of fiber-reinforced composites is predominantly a result of plastic micro-buckling of the fibers in an idealized inelastic matrix medium [2]. Other modes of failure that have been reported include fiber kinking, splitting, and delamination. Along the same lines, Kyriakydes et al. [5] have observed regularly spaced in-plane kink-bands with a width of ~1–1.5 mm during compression testing of the composites. In addition, Vogler and Kyriakides [6,7] have shown that kink-band formation is a post-buckling event and starts from a local imperfection in the sample. In a micromechanical study, Prabhakar et al. [8] have observed an interaction between the failure modes like kink-band and splitting, and concluded that mode II cohesive shear strength has a greater influence on this failure mode interaction. 

Despite the significant advancements in materials as well as design and manufacturing of the composites, ensuring a longer life for the blades has been a great challenge. Wind turbine system reliability is a critical factor for optimizing the costs [9]. Several collaborative efforts between industry and academia have gone into understanding and improving the reliability of wind turbine blades, with specific emphasis on flow characterization and manufacturing defects [10,11]. These and several other studies on failed wind turbine blades based on fiber-reinforced composite laminates have suggested that manufacturing flaws like voids, porosity, improper wetting of fibers with resin, misalignment of fibers, and bonding defects play a key role in their failure [11,12,13,14]. A 30%–50% drop in the compressive strength of the laminate has been observed even with a minor fiber misalignment of ~5° [15]. At higher off-axis angles (10° < *θ* < 15°), the compressive strength further dropped to 70%. Adams and Bell [16] studied two-layer out-of-plane waviness on a unidirectional ply at the middle of an eight-layered cross-ply laminate. They noted a ~36% reduction in compressive strength of the wavy laminate. Mandell et al. [17] studied the effect of fiber waviness with various glass and carbon fabrics used in wind turbine blades. They also noted a drop in compressive strength with an increase in the percentage of 0° layers containing waviness. Several other studies have also reported similar observations of reduced compressive strength in the presence of waviness defects [10,18]. Though there are many types of fiber waviness, in the present work, we will be focusing on the effect of an in-plane, through-thickness single sine wave with different severity levels under compression loading. Rather than degradation in material properties, the emphasis will be on the failure modes and mechanisms. This is important because limited research is available that combines both experimental and numerical approaches to explain the failure of these laminates.

## 2. Experimental Work

### 2.1. Materials and Processing

**Figure 1:**
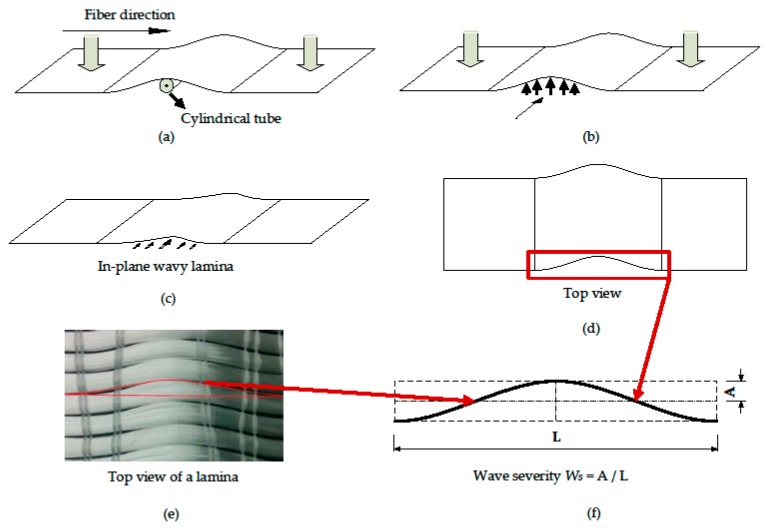
A scheme showing the procedure to induce an in-plane wave in a lamina: (**a**) inducing an out-of-plane wave with the help of a cylindrical tube, (**b**) out-of-plane fibers after the removal of the tube, (**c**) applying in-plane shear to make fibers in-plane, (**d**) a top view of the lamina, (**e**) preview of the in-plane wavy fibers, and (**f**) definition of fiber wave severity.

E-glass 0° (Wee Tee Tong UT800, Singapore) fabrics were used in this study along with epoxy resin as matrix material (Epikote RIMR—135 (Hexion^TM^, Kedah, Malaysia) as base and Epikure RIMR—137 (Hexion^TM^, Kedah, Malaysia) as hardener). This grade of resin is commercially used for wind turbine blade manufacturing and hence adopted in this study. An in-plane wavy laminate panel was manufactured by inducing a wave with the help of a cylindrical rod on each lamina of the unidirectional fabric. This whole process is shown schematically in Figure 1. As shown in Figure 1a, initially, out-of-plane fiber waviness was induced with a wavelength of ≈35 mm, and subsequently a uniform shear load was applied for ~5 to 10 min normal to the fiber direction and along the fiber fabric plane to render it in-plane (see Figure 1b). Each lamina undergoes the same process before stacking and curing. Different wave severities were achieved by changing the wave amplitude rather than changing the wavelength of the defect (due to size limitations for testing and analysis). An average wave height (twice the amplitude) of 0.7, 1.75, 2.45 and 5.25 mm were measured from each panel after the curing and final wave severity was calculated. 

The six layer ([0]_6_) panels were prepared with different wave severity levels using unidirectional glass fabrics. Each layer has an average thickness of 0.62 mm. The composite panel fabrication was done using Vacuum Assisted Resin Infusion Method (VARIM). The cure profile employed was 24 h at room temperature, followed by a post-curing step in an oven for 15 h at 60 °C. The fiber volume fraction of the cured panels was measured to be in the range of 0.52 to 0.57 based on ASTM D2584 [19].

### 2.2. Definition of Fiber Wave Severity

The definition of fiber waviness in the composites was first introduced by Joyce et al. [15,20] as follows: y=Asin(2πx/L) for a sinusoidal wave where the mean fiber position is parallel to the longitudinal fiber axis. A statistical approach followed with the help of computer-aided optical microscope to measure the wave amplitude (*A*) and wave length (*L*). The intensity of the fiber waviness was quantified with a term called fiber wave severity (W_s_), which is the ratio of *A* to *L* (see Figure 1f). After the curing of composite panels, amplitude and wavelength of the fiber waviness were again measured with the help of an optical microscope. The final fiber wave severity was taken based on the average values measured at different locations of the cured panel.

### 2.3. Compression Testing

**Figure 2:**
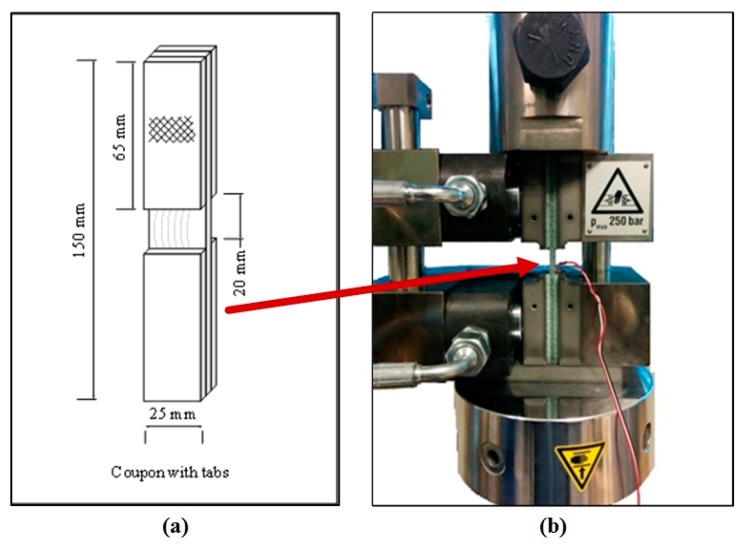
(**a**) Schematic of a typical compression test specimen, and (**b**) the actual HCCF set up with sample.

Compression tests were conducted as per ASTM D6641 standard [21] on a 100 kN servo-hydraulic Instron 8801 machine (Singapore) with Zwick Hydraulic Composite Compression Fixture (HCCF) (Singapore). A typical compression experiment specimen was 140 to 150 mm long and 13 mm wide with an unsupported gauge length of 13 to 20 mm. To avoid premature failure over the gage length, a smooth specimen edge and a sufficient surface roughness for the tab bonding was recommended [22]. The defect-containing specimens have an in-plane waviness defect at the gauge area. A width of 25 mm was considered for defect-induced specimens (see Figure 2) to minimize the percentage amount of discontinuous fibers due to waviness inclusion in the coupon. Compression test fixtures were selected based on combined shear and end loading [6,7]. The combined end and shear loads were applied hydraulically on the coupon at a constant crosshead speed of 1 mm/min. Compressive strains were measured using strain gauges (120 Ω) bonded on both sides of the coupon gauge area. The bonded strain gages on both sides of the gage part were connected to the data logger for the compressive strain measurement. During the test, failure progression was captured using a high-resolution video camera at the rate of 50 frames/s. Tests on each fiber wave severity specimen were repeated 5 times for getting statistically significant data.

## 3. Finite Element Model (FEM)

**Figure 3:**
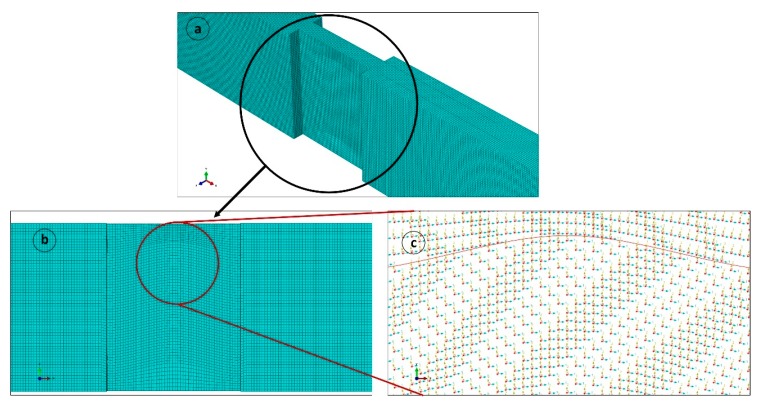
(**a**) Abaqus model with closely packed mesh, (**b**) mesh flow in the gage area along the waviness path, and (**c**) element orientation in the waviness region.

**Figure 4:**
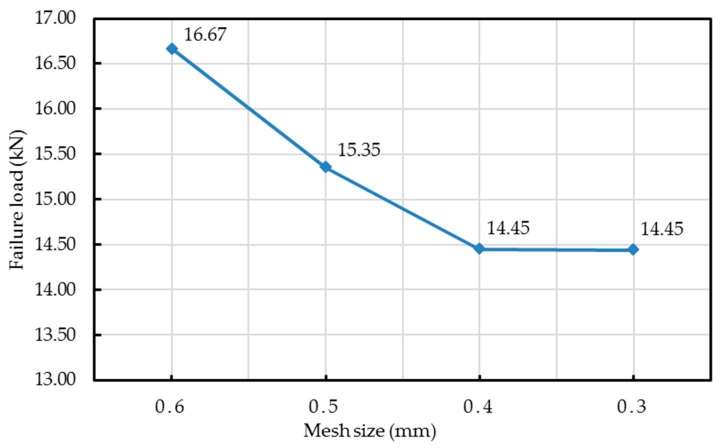
Mesh refinement for the wave induced model (wave severity = 0.075).

A 3D finite element model of the entire experimental specimen was built in Abaqus/Explicit for direct comparison with experimental results. The failure modes observed on unidirectional specimens without waviness defects were not detailed in this FEM analysis. A macro-mechanical model was used with an explicit dynamic solver at quasi-static loading rates. The plies were built using an 8-node brick element with reduced integration C3D8R and one element thickness for each ply. In the actual experiment, there was no damage to the tab material and the adhesive joint observed. Hence, tabs were modelled based on a high stiffness glass fiber reinforced polymer (GFRP) composite. The entire laminate with in-plane waviness was meshed in the gage section of the part geometry. Minimum building block in the model is lamina level, hence all the elements at the wavy regions were oriented through a discrete path (see Figure 3) that looked like the fiber waviness in the experimental samples. A mesh refinement study was conducted on the waviness-induced laminate until the change in predicted failure load was less than 1% (see Figure 4) and an optimum mesh size of 0.4 mm was selected. The thickness of the element was similar to the average ply thickness of 0.62 mm. The specimen model section was about 3.72 mm thick and 25 mm wide.

In general, the failure theories included in Abaqus (Max Stress and Max Strain, or Tsai-Wu and Tsai-Hill models) are not based on the individual constituents and result in poor prediction. Therefore, in this work, an Autodesk add-on for the simulation of composite analysis called Helius PFA (Progressive Failure Analysis) [23] was attached to the Abaqus model to predict the damage in the composite structures. This plug-in helps to incorporate the constituent (matrix and fiber) level failure initiation criteria like MCT (Multi continuum theory) based failure [24], Christensen [25], Puck [26] and LaRC02 [27] into the Abaqus software (Dassault systems, v6.13, SIMULIA, Johnston, RI, USA). Helius PFA uses separate material manager to include the composite lamina properties. Based on the input provided to the material manager, Helius PFA provides the constitutive relations for composite materials as per the required failure theory to the Abaqus input. As seen in Table 1, the lamina material properties were derived from the individual material properties input (fiber and resin properties) given to the material manager of Helius PFA.

In the current work, LaRC02 failure criterion (based on an improvement to Hashin’s model) was preferred as it combines the fracture plane concept of Puck [26] as well. This criterion identifies the fiber failure and matrix cracking in unidirectional composites (initiation and instantaneous damage progression) [26,28] based on the below mentioned constitutive relations. The uniaxial tensile strength (S_11_), compressive strength (-S_11_), and in-plane shear strength (S_12_) were obtained from the experiments conducted. The out-of-plane shear strength values S_23_ and S_13_ were considered equal to the in-plane shear value. The transverse parameters S_22_ and S_33_ were taken as √3 times of S_23_.

Individual plies were defined under a composite layup sequence with orthotropic non-linear elastic material properties based on experimental measurements. Experiments were conducted on the same material to determine the maximum tensile, compressive and shear strength properties. The detailed material properties used for the model are listed in Table 1, Table 2 and Table 3. Based on the LaRC02 failure criterion under uniaxial compression loading: (1) Fiber failure is further divided into (1.a) fiber compressive failure with matrix compression, and (1.b) fiber compressive failure with matrix tension. (2) Matrix cracking is again divided into (2.a) matrix cracking in tension, and (2.b) matrix cracking in compression. For the current model, due to the presence of fiber waviness imperfections in the cured laminate, it was assumed that majority of failure progression occurred due to fiber kinking. The fiber compression failure scenario was explained due to the collapse of fibers subjected to initial misalignment, leading to shear kinking and further extending to the supporting matrix [29,30].

(1) As mentioned earlier, under fiber compression $\sigma_{11}<0$, two stress states (matrix under compression and matrix under tension) were considered to evaluate the fiber failure [1].
  (1.a) For matrix compression ($\sigma_{22}^{m}<0$)
    (1) $$\mathrm{Failure}\;\mathrm{index}\;FI_{F}=\;\langle \frac{\left|\tau_{12}^{m}\right|+\eta^{L}\sigma_{22}^{m}}{S^{L}}\rangle$$
  (1.b) For matrix tension ($\sigma_{22}^{m}\geq 0$)
    (2) $$\mathrm{Failure}\;\mathrm{index}\;FI_{F}=\;{\left(\frac{\sigma_{22}^{m}}{Y^{T}}\right)}^{2}+{\left(\frac{\tau_{12}^{m}}{S^{L}}\right)}^{2}$$
(2) For the matrix compression failure criterion,
  (2.a) $\sigma_{11}\geq Y^{C}$
    (3) $$\mathrm{Failure}\;\mathrm{index}\;FI_{M}=\;{\left(\frac{\tau_{eff}^{T}}{S^{T}}\right)}^{2}+{\left(\frac{\tau_{eff}^{L}}{S^{L}}\right)}^{2}$$
  (2.b) $\sigma_{11}\leq Y^{C}$ at this stage the material is in a moderate biaxial compressive state and the condition is,
    (4) $$\mathrm{Failure}\;\mathrm{index}\;FI_{M}=\;{\left(\frac{\tau_{eff}^{mT}}{S^{T}}\right)}^{2}+{\left(\frac{\tau_{eff}^{mL}}{S^{L}}\right)}^{2}$$

where $\tau_{eff}^{mT}$ and $\tau_{eff}^{mL}$ are functions of the fracture angle α which will determine during the iteration.

$Y^{T}$ = Value of $\sigma_{22}$ at transverse tensile failure
$Y^{C}$ = Value of $\sigma_{22}$ at transverse compressive failure
$S^{L}$  = Absolute value of $\sigma_{12}$ at longitudinal shear failure
$S^{T}$  = Absolute value of $\sigma_{23}$ at transverse shear failure
α    = Angle of the fracture plane that maximizes the failure index (FI)
Subscript M represents the matrix and F represents the fiber.

Based on these constitutive equations of LaRC02 failure criteria, Abaqus identifies the failure initiation at individual integration points in the model. The results were interpreted based on the State Dependent Variables (SDV). SDV = 1.0, signifies no damage in both fiber and matrix and SDV = 2.0 signifies failed matrix. When SDV reaches 3.0 on a specified location, it was confirmed that both matrix and fiber failed in that area.

### 3.1. Damage Evolution

The damage evaluation process starts immediately after the damage initiated at any of the individual integration points. An instantaneous degradation method was followed for the stiffness degradation. The degradation ratio for both the matrix and fiber were predefined as user material constants (UMC) before the analysis. In here, UMC for matrix was set as 0.1 and for fiber, it was 1 × 10^−6^.

### 3.2. Boundary Conditions

**Figure 5:**
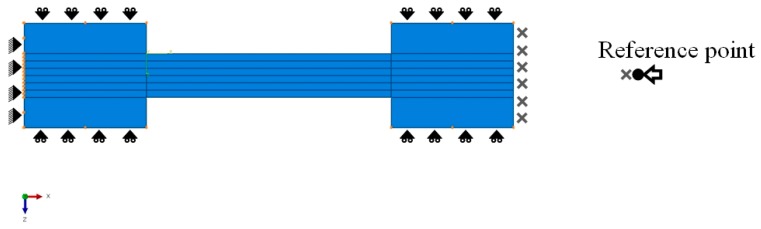
Boundary conditions at both ends of the sample.

In the model, one end of the specimen was fixed and a quasi-static displacement load was applied on the other end. Fully constrained boundary conditions were applied on the left (fixed) end and displacement boundary conditions were applied to the load direction by constraining the other directions to a reference point on the right end. The reference point and the right end of the model were bonded with an equation based directional constraint. So, the displacement given to the reference point would reflect to the right end face of the model. Figure 5 shows the 2D view of the trimmed model with 6 layers of ply material, tab bonding with boundary conditions at both ends and tab sides.

## 4. Results and Discussion

### 4.1. Effect of Waviness Defect on Compressive Strength

As listed in Table 4, the mean failure strength of the composites significantly decreases with increase in fiber wave severity. For instance, with a severity of 0.075, a 75% drop in strength is noted. Joyce and Moon [15] have reported a similar but linear trend of decreasing compressive strength with increasing (in-plane) fiber waviness severity. This has been attributed to the formation of kink-bands at the fiber misorientation sites in the wavy regions leading to a catastrophic failure. In the current work, though a catastrophic failure is observed with defect-free samples, with waviness defects, the failure is not catastrophic.

### 4.2. Mechanisms of Failure

#### 4.2.1. Unidirectional Laminates without Waviness Defects

**Figure 6:**
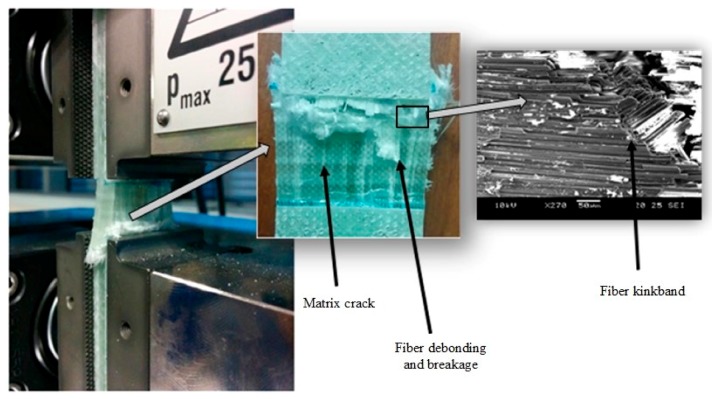
Catastrophic failure of waviness-free specimen under compressive loading conditions.

Optical and SEM observations of the failed samples reveal the presence of an angled fracture plane due to shear failure (Figure 6). Fiber strand debonding, micro-buckling and matrix cracking are also clearly visible in the gage area. These observations follow the traditional and expected mode of failure [31], but the sudden and catastrophic crushing of the gage area during testing has made it difficult to differentiate the various failure modes. Fiber micro-buckling is a result of shear instability process that occurs at higher strains in the matrix material due to plastic yielding. It is believed that during compression, the presence of fiber misalignment imperfection from the longitudinal direction results in the formation of kinks in a localized region. This ultimately leads to the formation of fiber kink-bands followed by compressive kinking failure [32]. However, it is important to note that kinking stresses are very sensitive to fiber misalignment. Even a misalignment angle in the range of 0.8–2.3° was enough to cause kinking [33]. Moreover, with misaligned fibers, kinking stresses are reportedly 25% of the elastic micro-buckling stresses of composites.

#### 4.2.2. Unidirectional Laminates with Waviness Defects

**Figure 7:**
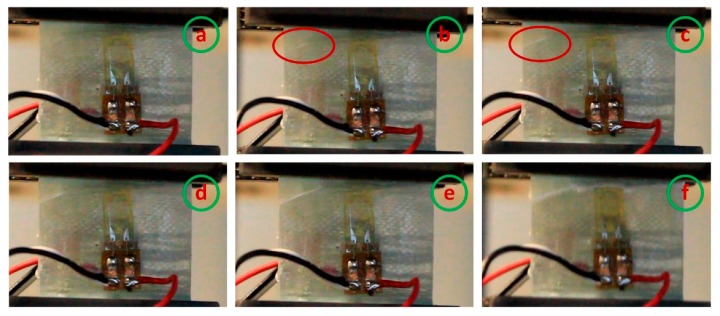
Sequence of crack propagation before complete failure in sample A1: (a) at the start of the test, (b) crack initiation (region within the red color oval), (c) crack propagation towards the free edge, (d) & (e) widening of the crack, and (f) failure.

**Figure 8:**
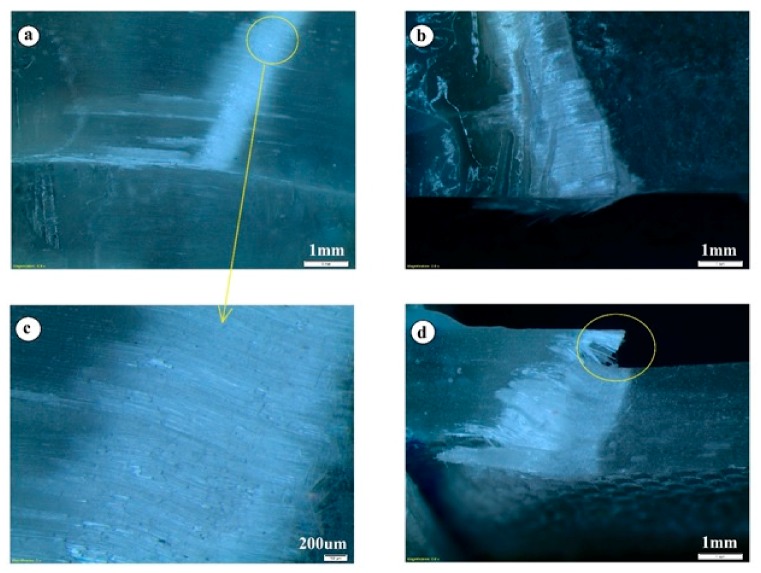
(**a**) Fiber kinking and fiber splitting along the wavy fiber direction in sample A3, (**b**) kink-band view at the free edge, (**c**) magnified fiber kinking view and (**d**) fiber breakage.

As compared to defect-free specimens, the failure progression of the wavy specimens is slow and gradual (particularly, A4 with a wave severity of 0.075). Figure 7 shows a few selected snapshots of crack initiation and progression from the video of the test captured with a high-speed camera operated at a frame rate of 50 frames/s. As discussed earlier, axial compression failure of unidirectional composites occurs by plastic kinking in the presence of fiber misalignment sites along with plastic shear deformation in the matrix. Figure 7 shows that the failure initiated with a visible fiber kinking followed by an inclined shear crack across the fiber direction in the wavy area and ultimately results in fiber strand splitting. Previously, it has been noted that glass fibers fail in compression by longitudinal splitting when the uniaxial strain in the composite equals the intrinsic crushing strain of the fibers [4]. Nevertheless, the gradual kink-band formation and the resultant fiber kinking failure mode are more evident at higher fiber wave severities (A3 and A4 samples). For example, Figure 8 shows the failure progression in A3 with wave severity 0.035.

A clear transition in the failure mode is evident in composites with and without waviness defects. Moreover, the crushing phenomenon during the failure is not seen at higher fiber wave severity levels. However, audible crushing and/or knocking sounds are heard in composites with a wave severity 0.025 (Sample A2, misalignment angle ~5.7°) and below. Thus, a wave severity of 0.025 seems to be a transition point in failure mode. The width of the kink-band increases with increasing wave severity. The kink-band width changed from ~0.1–0.12 mm in A0 sample to ~1.5–2 mm in A3 sample.

### 4.3. Experimental Observations versus Abaqus Model

**Figure 9:**
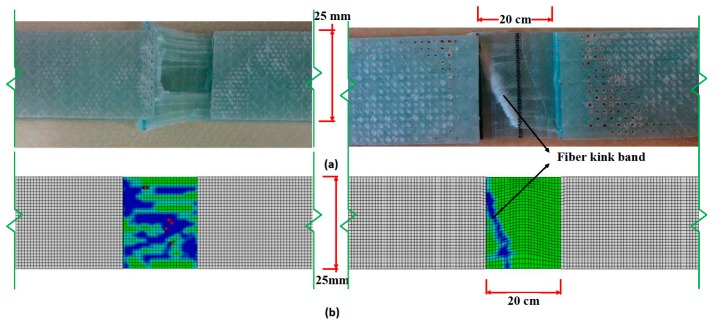
(**a**) Experimentally failed specimens and (**b**) model prediction of both defect-free and waviness defect-containing samples.

**Figure 10:**
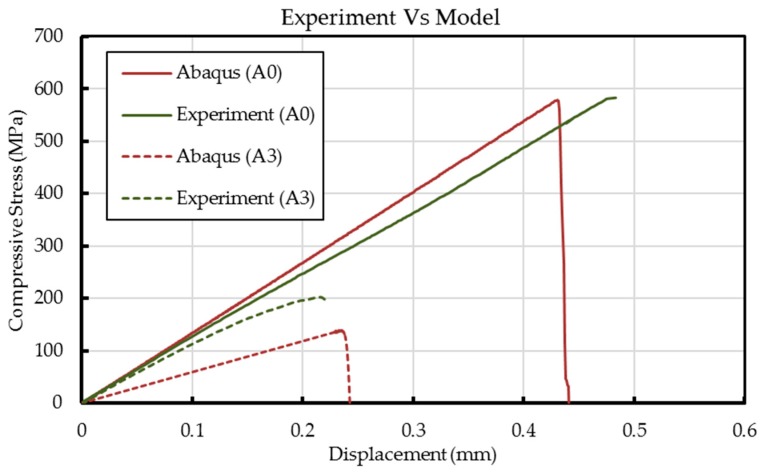
Experimental data and model predictions of compressive stress vs. displacement behavior of A0 and A3 samples.

As mentioned earlier, a complete specimen model had been developed for direct comparison of experimentally observed failure outcomes. Based on the LaRC02 failure criterion, experimental observations and results fit well with model predictions (Figure 9 and Figure 10). The advantage of using LaRC02 failure criterion is that under compression condition, the fiber failure is caused by shear kinking and damage of the matrix. The misalignment of the fibers due to the waviness defects drives the failure towards kink-band formation. As discussed earlier, a similar failure mode trend is seen during the experiment. 

In Figure 9b, the elements with blue color have an SDV value of 3 and above, meaning both fiber and matrix are damaged in this region. As kink-band formation is believed to be controlled by the misalignment of fibers and the shear strength of the matrix, a shear response analysis has also been conducted. It is found that fiber waviness has little influence on shear strength other than the non-linear shear response. This once again suggests that fiber waviness does not affect the fiber/matrix interfacial strength, which is a key property influencing compressive strength and kink-band formation during failure [6]. Hence, it can be confirmed that waviness is the major reason for the kink-band mode of failure.

Based on the Abaqus model (Figure 9b), it is evident that the damage (crack) has initiated at the middle of the gage length. However, cracks have appeared at much lower loads in the waviness defect-containing samples. The discontinuity in fibers (at the top and bottom of the waviness containing samples) are the weak links in the system and the failure propagation diverted towards those areas. In the actual structures, fiber discontinuity would not be present along with the fiber waviness defect unless it were a ply drop situation. In A3, kink angles are measured in the range of 110° to 118°, and from the model, the angle is ~116°. Hence, we can conclude that the model prediction is closer to the experimental findings.

**Figure 11:**
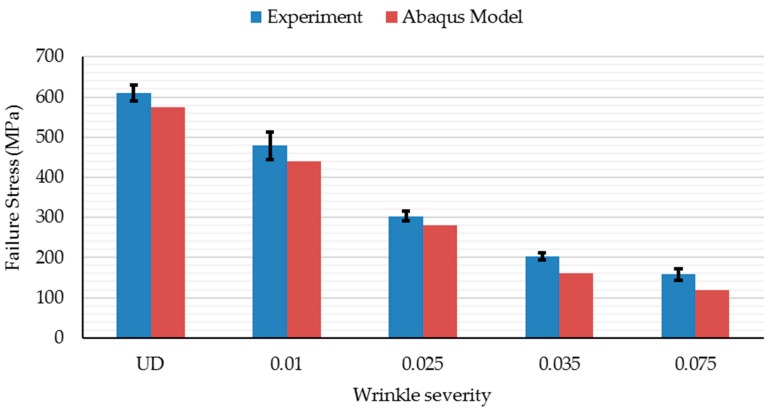
Comparison of compressive strength of GFRP laminates with different wave severity.

The Abaqus model is a lamina level macro-mechanical model, and the drop in the stiffness properties of laminate purely depends on the elemental stiffness. Hence, a change in element orientation in the wavy region results in the reduction of elastic modulus in the model. However, in the experiment, the fiber and matrix are individual constituents and the fiber waviness alters only the fiber properties. This is possibly a reason for the small reduction observed in elastic modulus of the laminate when compared to the model. For the wavy specimen with a wave severity of over 0.025, a difference greater than 5% in the predicted elastic modulus is observed. Unlike the experimental data that showed non-linear behavior, the Abaqus model failed to predict the slight non-linearity in the stress-displacement curves of waviness defect containing samples. Even the observed fiber breakage and matrix cracking (see Figure 8) could not be predicted with the FEM model. To sum up, Figure 11 shows the effect of manufacturing-induced fiber waviness on the compressive strength of GFRP laminates. As shown, a single through-thickness wave of severity 0.025 is enough to bring about a reduction of up to 50% in the compressive strength.

## 5. Conclusions

Compression tests were performed on unidirectional GFRP laminates with induced, through-thickness, in-plane waviness defects. It was noted that both the stiffness and compressive strength of the laminates decrease significantly with increasing severity of waviness defects. The drop in compressive strength was found to be ~75% at a wave severity level of 0.075. Failure micro-mechanisms revealed that kink-band formation was the major mode of failure in these samples. Kinking was due to micro-buckling because of waviness/discontinuity in fibers. As the wave severity increased, the failure mode transited from a sudden crushing failure to a clear visible kink-band formation.

A finite element model based on an improved (LaRC02) failure criterion has been developed, which agrees well with the experimental response and failure modes within the reasonable limit in the case of waviness defect-containing samples. Compared to many other existing failure criteria, the current improved Hashin’s failure criterion successfully predicted the fiber kinking failure of waviness-induced samples under compression loading conditions. This confirmed the involvement of remotely misaligned fibers in fiber collapse and kink-band formation leading to further matrix damage. Though the model was successful in predicting the failure behavior with different wave severity levels, differences in elastic modulus between experiments and model were obvious at a wave severity of 0.025 and above.

As it is difficult to avoid fiber waviness defects while using the current manufacturing techniques for composite blade structures, it is important to take additional care while stacking the fiber fabrics. This could help in minimizing their appearance or in reducing their severity. 

## Figures and Tables

**Table 1:** Constituent elastic properties used in the Abaqus model (based on material data sheet).

Fiber		Matrix	
**Young’s Modulus** $E_{f}$ **(GPa)**	**Poisson’s Ratio**	**Shear Modulus** $G_{12}$ **(GPa)**	**Poisson’s Ratio**
73	0.24	1.2	0.35

**Table 2:** Lamina strength properties used in the Abaqus model (Experimentally determined).

Ultimate Tensile Strength $X_{T}$ (MPa)	Ultimate Compressive Strength $X_{C}$ (MPa)	Ultimate Shear Strength $G_{12}$ (MPa)
728	630	50

**Table 3:** Comparison of lamina material properties from experiment and model.

Elastic properties	$E_{11}$ (GPa)	$E_{22}$ (GPa)	$G_{12}$ (GPa)	$\nu_{12}$
**Based on Autodesk Helius PFA**	41.4	9.99	3.58	0.27
**From experiment**	41.4	10.29	2.78	0.28

**Table 4:** Compression test results of composites with and without waviness defects.

Sample	Wave Severity (*W_s_*)	Mean Failure Strength (MPa)	Standard Deviation (MPa)
**A0 (unidirectional)**	0	614	19.61
**A1**	0.01	479	34.5
**A2**	0.025	303	12.25
**A3**	0.035	203	13.16
**A4**	0.075	158	14.40
